# Experimental Investigation and Modeling for the Influence of Adding Date Press Cake on Drinkable Yogurt Quality

**DOI:** 10.3390/foods12061219

**Published:** 2023-03-13

**Authors:** Nashi K. Alqahtani, Tareq M. Alnemr, Abdullah K. Alsalem, Mutlaq M. Alotaibi, Maged Mohammed

**Affiliations:** 1 Date Palm Research Center of Excellence, King Faisal University, Al-Ahsa 36362, Saudi Arabia; nalqahtani@kfu.edu.sa; 2 Department of Food and Nutrition Sciences, College of Agricultural and Food Sciences, King Faisal University, P.O. Box 400, Al-Ahsa 31982, Saudi Arabia; talnemr@kfu.edu.sa (T.M.A.);; 3 Department of Dairy Science and Technology, Faculty of Agriculture (El-Shatby), University of Alexandria, Alexandria 21545, Egypt; 4 Department of Agricultural and Biosystems Engineering, Faculty of Agriculture, Menoufia University, Shebin El Koum 32514, Egypt

**Keywords:** dairy alternatives, design expert, food quality, texture profile analysis, sensory evaluation, prediction model, food waste upgrading

## Abstract

The extraction of date syrup produces a large quantity of by-product known as date press cake (DPC). This study aimed to utilize valuable ingredients of the DPC by adding 0 (Control), 2, 4, and 6% (g/100 g) of its powder to drinkable yogurt before fermentation. The physicochemical properties, texture profile, and sensory evaluation of the treated DPC-based drinkable yogurt (DPC drinkable yogurt) were measured after fermentation and 5, 10, and 15 days of storage at 4 °C. The modeling of the most critical quality attributes, i.e., pH, acidity, syneresis, water holding capacity (WHC), viscosity, and color difference (ΔE), was conducted to predict their values based on the DPC percentage and storage period. The DPC drinkable yogurt’s total solids, protein, and fat ranged between 11.19–11.83, 3.10–3.42, and 2.26–2.34%, respectively. Adding 2–6% DPC slightly increased the pH of DPC drinkable yogurt and decreased its acidity (*p* > 0.05) during storage. Increasing the DPC percent in DPC drinkable yogurt decreased the syneresis value, and WHC increased during storage. The color parameters and viscosity of DPC drinkable yogurt recorded the highest value at the end of the storage period for all treatments and increased steadily with the increase in DPC. The evaluation of the prediction models indicated that the predicted values were close to the actual experimental values for pH (R^2^ = 0.779), acidity (R^2^ = 0.973), syneresis (R^2^ = 0.961), WHC (R^2^ = 0.989), viscosity (R^2^ = 0.99), L* (R^2^ = 0.919), a* (R^2^ = 0.995), b* (R^2^ = 0.922), and ΔE (R^2^ = 0.921). The textural analysis indicated that increasing the concentration of DPC in the DPC drinkable yogurt increased hardness (g), springiness, cohesiveness, and gumminess and decreased adhesiveness and resilience during cold storage. The evaluation of sensory acceptance during the cold storage of the DPC drinkable yogurt was conducted by 30 expert panelists. Each panelist received four cups of 10 mL drinkable yogurt treatments at 5–10 °C. The evaluation results indicated that adding 2% of DPC was closest in overall sensory acceptability to the control sample (*p* < 0.05). This study revealed the potential use of DPC in drinkable yogurt as a natural, functional, and low-cost ingredient to improve the fiber content, physicochemical properties, and overall acceptability. Therefore, the fermented DPC-based yogurt drink has the potency to be a practical, value-added, and novel alternative to dairy-based yogurt.

## 1. Introduction

Food functionalization is one of the ever-growing markets, which require new bioactive ingredients. In addition, the bioactive components can be used to develop innovative functional products [1]. However, with the limited natural sources of functional compounds to achieve this effect, most productive research studies have turned to natural alternatives to maintain the increasing demand for the ingredients added to those foods. Therefore, the world is now turning to the exploitation of biological agricultural wastes, which constitute a great environmental burden [2]. However, agricultural waste ingredients contain significant active functional additives, such as dietary fiber, sugars, proteins, phenols, antioxidants, anti-carcinogens, pressure reducers, and cholesterol. In addition, these products are produced without additional production costs [2,3]. 

Furthermore, recent research studies have shown that agricultural by-products or agricultural wastes and their extracts can be successfully incorporated as functional ingredients for developing new food products [4,5,6]. For example, the extraction of date syrup (Dips) produces large quantities of by-products known as date press cake (DPC), which contain high-fiber waste retained after filtering the Dips. DPC is a major waste and an environmental burden due to its high content of sugars and moisture. DPC is regarded as one of the functional raw materials, which can be developed and used as an added value to produce healthy foods [7]. The dips industry generates about 17–28% of DPC, often used in animal feeds as a filler [8] or disposed of in burial or sewage, which constitutes environmental pollution [9]. The lack of optimal use of that by-product may be due to the lack of chemical composition, health benefits, and knowledge of its nutritional applications when used in foods. It was only previously used as a raw material in the production of active carbon or feed ingredients [8]. Research studies indicated that DPC of the Khalas variety of date fruit contains the essential constituents (7.16% moisture, 2.82 ash, 2.65% protein, 0.81% fat, 86.6% carbohydrates, 8.5% fructose, 5.6 % glucose), and soluble fiber content was 6.5%. The insoluble fiber was 49.99%, while DPC per 100 g contained 194.39 mg calcium, 10.73 mg iron, 443.38 mg potassium, and 97.41 mg magnesium. Its water absorption was 1.98 g/g, and fat constituted 0.67 g/g. The emulsifying capacity was 56.17. The emulsifying stability was 71.46% [9,10].

The consumer orientation emphasizes healthy foods, which contain functional products. Dairy products are a fertile field in terms of the possibility of developing them by integrating many available added-value ingredients with milk products, such as yogurt. Yogurt is already considered a healthy food because it contains viable bacteria but does not contain dietary fiber compounds [11,12]. Yogurt is an exceedingly consumed dairy food globally, renowned for its health benefits, nutritional value, and digestibility [13]. Yogurts are vastly consumed across the globe and are produced by fermenting different types of milk with bacteria, such as *Streptococcus thermophilus*, *Lactobacillus bulgaricus*, resulting in a product with creamy characteristics and a slightly acidic taste [14]. Yogurt is becoming more vogue due to probiotics, higher digested nutrients, gel-like structure, taste, and mouthfeel [15]. 

The most important features for the consumer acceptance of yogurts are texture and firmness, related to viscosity and stability. In addition, adding other materials, such as pectin, gelatin, inulin, or dietary fiber, may enhance some sensory acceptance of yogurt and decrease syneresis [16]. Therefore, the possibility of improving and modifying the characteristics of those products to avoid excessive use of additives is achieved for the same purpose. Furthermore, yogurt is one of the most famous milk products fermented with lactic acid bacteria, with a wide production and consumer acceptance of it and its health benefits. Therefore, it was one of the essential milk industries to develop, especially for enhancing it with upgraded agricultural waste ingredients [17]. In addition, enriching yogurt with dietary fiber sources is of growing interest in the creation of functional foods with health benefits, perfecting their functionality, and enhancing their nutritional value [18]. 

The quality attributes of yogurt that can be controlled with stabilizers or fibers include acidification, sensory aspects, gel structure, and syneresis [19,20,21]. In addition, such control allows for modifying the viscosity and lowering syneresis [21,22]. The “clean label” trend popular among consumer producers encourages the use of natural plant materials for stabilization purposes, the presence of which in a product does not raise any health controversies or concerns [23]. The results of some studies indicated the possibility of combining yogurt with vegetable fiber residues, such as date fruit, mango peel powder, citrus peel, persimmon and its powder, apple peel powder, tomato pomace, apricot press cake, sweet lupine husks, potato, cranberry fiber, and others [6,24,25,26,27,28,29]. This study aimed to investigate the effect of DPC powder addition on drinkable yogurt quality attributes before fermentation, model the influence of DPC addition and storage time on the most important physicochemical properties, and evaluate the textural and sensory properties of the fermented DPC-based yogurt drink.

## 2. Materials and Methods

### 2.1. Materials

DPC of date fruit (Khalas cv.) was obtained from Aldahaby Dates Factory, Al-Ahsa, Saudi Arabia. The DPC samples were dried under vacuum at 48 °C in an electric vacuum drying oven (LVO-2041P, Daihan Labtech Co., Ltd., Namyangju-si, Gyeonggi-do, Korea) for 72 h. They were then ground and sifted to obtain granules of 250 μm [30].

The drinkable yogurt was manufactured at the Agricultural Research Station pilot plant of King Faisal University. The freeze-dried starter culture used to ferment the yogurt was YC-X11 (Chr. Hansen company, Hørsholm, Denmark), containing *Lactobacillus delbrueckii subsp. Bulgaricus* and *Streptococcus thermophiles*. Cow’s milk was prepared in equal quantities for each treatment. The pH and fat of the cow’s milk used were 6.76 ± 0.04 and 2.26 ± 0.05 g/100 g, respectively. The DPC powder was added to T_0_, T_1_, T_2,_ and T_3_ with concentrations (g/100 g) of 0, 2, 4, and 6%, respectively. Then, the pasteurization process was performed at 90 °C for 10 min, followed by the sudden cooling process to 40 °C. According to the starter data sheet, a starter culture (50 units) was added to the previous treatments, then incubated at 42 °C for 3 h (fermentation complete). Finally, the fermentation was stopped by cooling at 4 °C. The measurements were taken immediately after fermentation and after 5, 10, and 15 days of storage at 4 °C [31].

### 2.2. DPC, Milk, and DPC Drinkable Yogurt Analysis

All measurements were carried out in three replicates for the milk used, DPC, and DPC drinkable yogurt treatments. Total solids (TS), protein, fat, ash, and pH were determined for the milk used for yogurt preparation by Lactoscan Funke-Gerber D-12105, Berlin, Germany. DPC drinkable yogurt treatments were analyzed in terms of total solids, protein, and fat, while DPC was analyzed in terms of moisture, protein, fat, ash, crude fiber, and water binding capacity. The solubility and color parameters were determined according to the standard methods of AOAC [32]. Meanwhile, the moisture, fat, ash, and dietary fibers were estimated by the gravimetric method No. AOAC 934.01. Kieldahl measured protein No. AOAC 976.05, and the obtained values were expressed as total nitrogen multiplied by 6.38 and 6.25 to obtain the total protein content in DPC drinkable yogurt treatments and the DPC. Fully automated grude and detergent fibre analysis (Fibertec^TM^ 8000, FOSS, Hilleroed, Denmark) was used to determine dietary fiber. The total carbohydrates were calculated mathematically (100 – (moisture% + fat% + protein% + ash%). The changes in chemical characteristics were tracked by analyzing the DPC drinkable yogurt treatments immediately after fermentation and cooling and then after 5, 10, and 15 days of storage at 4 °C in terms of each of the following.

#### 2.2.1. pH and Acidity

pH was measured with a Thermo Orion 3 Star pH Benchtop Meter (Fisher Scientific, Instruments, Pittsburgh, PA, USA), which was calibrated with a pH 4.00 and 7.00 buffer solution (Thermo Fisher Scientific, Waltham, MA, USA). Titratable acidity as a percentage expressed as lactic acid was also estimated according to the method described in AOAC [32].

#### 2.2.2. Syneresis, Water Holding Capacity, and Viscosity

Syneresis or whey separation was determined according to the method described by Cichońska et al. [33] using a centrifuge ((Hermel-Z233 M-2, Hermle Labortechnik GmbH, Wehingen, Germany). The 40 g samples were mixed and centrifuged at 4 °C and 16,125× *g* (× g is times gravity) for 20 min. The relationship between the centrifuge speed in revolutions per minute (RPM) and relative centrifugal force (RCF) was calculated as follows:

(1) $$g=\left(1.118\times 10^{-5}\right)R\;S^{2}$$

where g is the relative centrifugal force (RCF), R is the rotor radius in centimeters, and S is the centrifuge speed in RPM.

After complete expulsion, the separated serum was poured and weighed. The syneresis values of three replicates for each treatment were calculated as a percentage according to the following equation:

(2) $$S=\frac{m_{1}}{m_{2}}\times 100$$

where *S* is the syneresis percentage, $m_{1}$ is the mass of the separated serum after centrifugation in grams, and $m_{2}$ is the initial mass of the yogurt before centrifugation in grams.

Water holding capacity (WHC) values of the yogurt treatments were estimated according to the method described by Feng et al. [34]. First, a yogurt sample (10 g) is centrifuged for the three replicates at 1500 × g for 10 min; then, the filtrate is poured, and the precipitate is weighed. Then, WHC is calculated according to the following equation:

(3) $$WHC=\frac{m_{ys}-m_{s}}{m_{ys}}\times 100$$

where WHC is the water binding capacity (%), $m_{ys}$ is the mass of the yogurt sample, and $m_{s}$ is the mass of the sediment.

The viscosity values were estimated according to the method described by Cichońska et al. [33] using rotary viscometer LV DV-II+Pro (Brookfield Engineering, Middleboro, MA, USA) at 4 °C and a spindle (S64) with a rotation of 100 rpm by applying a constant shear speed (0.05 s^−1^). The readings were taken in the 15^th^ second of measurement. Three replicates of yogurt treatments were taken in centipoise units (cP).

#### 2.2.3. Color Parameters

The color characteristics of the drinkable yogurt treatments were measured according to the method described by Hunter and Harold [35] using the Hunter Lab color meter (Hunter Associates Laboratory Inc., Reston, VA, USA). The Hunter Lab color meter was calibrated before the measurements with black and white plates, where the reading was taken in (L*, a*, b*). The value of L* indicates the extent of lightness or luminance/darkness, ranging between the value of black and 100 for white. a* value expresses redness/greenness; the positive value of redness falls into negative greenness values. b* expresses yellowness/blueness and the positive values for yellow and negative for blueness. L* (brightness, 100 = white, 0 = black), a* (+, red; −, green), and b* (+, yellow; −, blue). The color difference (∆E) was calculated based on the International Commission on Illumination (CIE) lab using the following equation:

(4) $$\Delta E=\sqrt{{\left(L_{2}^{*}-L_{1}^{*}\right)}^{2}+{\left(a_{2}^{*}-a_{1}^{*}\right)}^{2}+{\left(b_{2}^{*}-b_{1}^{*}\right)}^{2}}$$

where ∆E is the color difference of the fruit, L* is fruit lightness, a* is greenness–redness, b* is blueness–yellowness.

#### 2.2.4. Texture Profile Analysis (TPA)

Texture profile analysis (TPA) features, which include hardness, adhesiveness, springiness, cohesiveness, gumminess, and resilience [36], were assessed by a double stress test using a texture analyzer (model: TA.XTplusC, Stable Micro Systems Ltd., Godalming, UK). Before the TPA analysis, the test samples were left at 10°. A 25 mm diameter perplex cylindrical probe was used to measure the textural profile of the yogurt samples at 10 ± 0.5 °C. The TPA analysis was performed by compressing twice using the probe for 10 mm penetration. In the first stage, the samples were compressed, and the probe’s speed was fixed at 5 mm/s during the samples’ pretest, compression, and relaxation. The load cell was 5 kg, and the trigger force was 0.1 N. The typical textural profile (force–time) curve was obtained with one complete run. The hardness, adhesiveness, springiness, cohesiveness, gumminess, and resilience of yogurt samples were calculated by the software included with the texture meter used.

### 2.3. Sensory Evaluation

Sensory attributes were assessed by 30 panelists (10 women and 20 men, aged 20 to 60 years) according to the method described by Wang et al. [37]. The acceptance test was conducted under the illumination of the sensory evaluation room maintained at 25 °C in the Department of Food and Nutritional Sciences, College of Agricultural and Food Sciences, King Faisal University. Sensory evaluations were carried out by 30 professional panelists, including teaching staff of the food science and nutrition department, university dairy pilot plant staff, and selected staff of dairy companies in Al-Ahsa 31982, Saudi Arabia. Each panelist received four cups of 10 mL drinkable yogurt treatments at a temperature of 5–10 °C, a sensory assessment sheet, and a water bottle for mouthwash provided between each sample assessment. A 5-point hedonic scale (1 = I don’t like it, and 5 = I really like it) was used. Samples were evaluated based on color, texture, flavor, and overall acceptability [38,39].

### 2.4. Statistical Analysis 

The results were statistically analyzed through the SAS program in four analyses during the storage period (0, 5, 10, and 15 days) of the prepared drinkable yogurt treatments (0, 2, 4, and 6% DPC) following a randomized complete block design. Duncan’s multiple range test (MRT) was used to determine the variance between treatments within the 0.05 level of significance, where the averages in the same columns with capital letters denote a significant difference within the level of significance (*p* < 0.05). Design Expert software (DX Version 13, Stat-Ease, Inc., Minneapolis, MN, USA) was used to graphically analyze the experimental data and model the influence of DPC addition and storage time on the physicochemical properties of the DPC drinkable yogurt.

## 3. Results and Discussion

### 3.1. Physicochemical Analysis of the Milk Used and DPC

**Figure 1:**
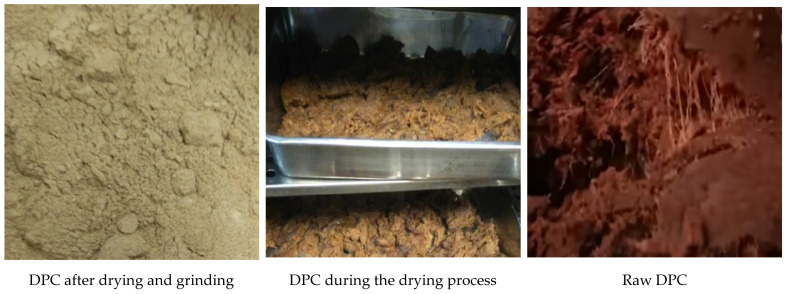
The appearance of DPC as raw material during drying and after drying and grinding.

As the results reveal in Table 1, the total solid, protein, fat, and ash contents (g/100 g) of the cow’s milk used in preparing yogurt drink treatments were added to the DPC. Additionally, DPC contents (gm/100 g) of moisture, total protein, fat, ash, crude fiber, total sugars, water holding capacity (WHC), solubility, pH, and color determinants were listed. The values of color are consistent with the shape appearance of the DPC used during drying and after grinding (Figure 1). 

The results of Khalas DPC analysis agreed with Al-Farsi et al. [40], d the DPC protein of Omani varieties ranged from 3.62 g/100 gm in Al- Shahal DPC to 5.23 g/100 gm in Mabseeli DPC. In contrast, the fat content ranged from 5.02 g/100 g in the Mabassili DPC to 5.90 g/100 g in the Um-sellah DPC. The dietary fiber ranged between 77.75 and 80.15 g/100 g, respectively. On the other hand, Hashim and Khalil [41] recorded the chemical compositions and physicochemical properties of Lulu, Khalas, and Barhi DPC, where the moisture content range was 8.73, 7.16, 6.14; ash—2.15, 2.82, 2.98; protein—2.18, 2.56, 3.09; fat—1.04, 0.81; fat—0.95; and carbohydrates—85.9, 86.56, 86.84, respectively. The study also recorded the values of colorimetric parameters for the same varieties in terms of the extent of whiteness or luminance/darkness L*, redness/greenness a*, and yellowing/blueness b*, where the values of L* were 48.64, 55.51, 54.76; the values of a*—7.63, 8.02, 7.34; and b*—17.78, 18.33, 18.22, respectively. The study also recorded the water holding capacity (WHC), which reached (g/g) 1.96, 1.98, and 2.0, respectively. Majzoobi et al. [42] mentioned that Iranian Shahani DPC had 13.37% moisture, 4.92% fat, 6.35% protein, 11.74% crude fiber, and 79.06% carbohydrates. The Khalas DPC contained 20.5% moisture, 72.7% carbohydrates, 3.12% protein, 0.31% fat, and 1.72% ash [9,10]. A study by Sheir [7] recorded Egyptian Saidi DPC’s contents, with moisture content of 6.11%, fat of 5.12%, protein of 7.4, crude fiber of 12.38%, ash of 2.78%, and carbohydrates of 66.21%.

### 3.2. Physicochemical Analysis of the Treated DPC Drinkable Yogurt

#### 3.2.1. Total Solids, Protein, and Fat of DPC Drinkable Yogurt

DPC drinkable yogurt treatments include the T_0_ treatment (the control sample free of DPC), while T_1_, T_2_, and T_3_ treatments contain 2%, 4%, and 6% (g/100 g) of DPC, respectively. The results in Table 2 show that the percentages (g/100 g) of total solids, protein, and fat for T_0_ were 11.19, 2.97, and 2.26; T_1_—11.83, 3.10, and 2.34; T_2_—12.92, 3.3, and 2.3; and T_3_—13.45, 3.42, and 2.35, respectively. Significant increases (*p* > 0.05) in total solids for DPC drinkable yogurt treatments due to the solid contents of DPC and the slight increases in fat and protein are due to the few ranges of DPC. These results agree with the studies, which supplemented yogurt with different sources of dietary fiber [11,43,44,45].

#### 3.2.2. pH and Acidity of DPC Drinkable Yogurt

**Figure 2:**
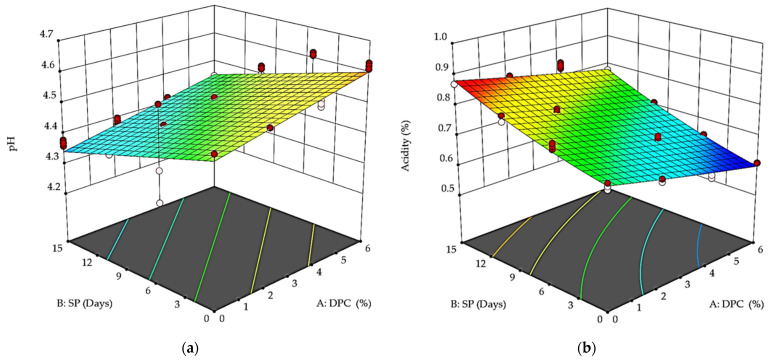
Effect of DPC addition percent (A: DPC) and storage period (B: SP) on pH (**a**) and acidity (**b**) of DPC drinkable yogurt treatments during storage period at 4 °C.

Increasing the percentage of DPC between 2 and 6% caused slight increases in pH (Figure 2a) and a slight decrease (*p* > 0.05) in acidity (Figure 2b). Generally, storage over 14 days affected the pH and acidity values, as the pH values decreased, and the acidity increased. The pH values for T_0_, T_1_, T_2_, and T_3_ were 4.51, 4.53, 4.55, and 4.63 at the beginning of the storage period, while they were 4.37, 4.39, 4.41, and 4.43, respectively, at the end of the storage period. The acidity of T_1_ had the greatest value and that of T_3_ the lowest value at the beginning and end of storage compared to the control sample, which recorded 0.61 and 0.76% at the beginning of storage and 0.77 and 0.81% at the end of storage, respectively. Compared to the control sample, with increasing the DPC addition, the pH values increased, and the acidity ratios decreased with the increase in DPC concentrations. However, with the progression of the storage period, the pH values decreased, and the acidity percentages increased. This increase in pH with DPC addition can be attributed to its ability to retain water and thus dilute the concentrations of lactic acid and other organic acids produced by the starter culture. This may increase the pH, especially in yogurt fortified with dietary fiber [29,46]. The acidity estimation results for all treatments were kept at 0.87%, as recommended for yogurt [47]. The pH values of all yogurt treatments increased with increased DPC and water absorption from its fibers. Some research studies have reported that adding different dietary fiber sources affected the pH and acidity of fortified yogurt [44,48]. On the other hand, not all the fruit peels or their residues affected yogurt fermentation. No differences were recorded in the acidity degree of the yogurt to which papaya peel flour was added during storage [49]. At the same time, the acidity of yogurt containing passion fruit peel powder was much higher than in the respective controls, which is the behavior expected by the metabolism of lactic acid bacteria [50]. Factors such as total soluble solids, storage temperature, and additives can also reduce the pH of yogurt [51] due to post-acidification and increased activity of lactic acid bacteria. Adding fruits or pulp to yogurt stimulates starter culture bacteria to increase acidity and lower the pH compared to some peels or fibers of these fruits [48,52].

#### 3.2.3. Syneresis, WHC, and Viscosity of DPC Drinkable Yogurt

**Figure 3:**
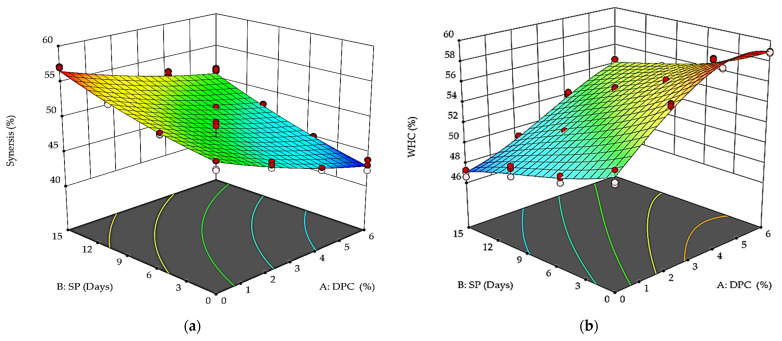
Effect of DPC addition percent (A: DPC) and storage period (B: SP) on syneresis (**a**) and WHC (**b**) of DPC drinkable yogurt treatments during the storage period at 4 °C.

The results in Figure 3a indicate that the storage time and DPC addition slightly affected the syneresis values, which decreased with the increase in DPC addition and during the storage period. The highest ability to retain whey (the lowest degree) was noted immediately after fermentation. T_0_ was significantly higher for syneresis (*p* < 0.05) than T_1_, T_2_, and T_3_ during storage. Syneresis values increased during storage time, but the rate of increase was lower for DPC drinkable yogurt treatments. The rate ranged during the beginning and end of the storage period between 50.26 and 57.06 for the control sample, while for the T_1_, T_2_, and T_3_ samples, the ranges were 48.03–53.26, 45.03–52.23, and 43.1–51.33, respectively. Thus, the increased amounts of DPC added reduced the syneresis. T_3_ was the lowest syneresis value (*p* < 0.05); this decrease may be attributed to an increase in total solids, as mentioned by Mahdian and Tehrani [53]. Recent studies [6,11,24,54,55] reported that fortification of yogurt with different dietary fibers supports the viscosity and thickening properties of the yogurt gel. The decrease in syneresis in the DPC drinkable yogurt treatments may be attributed to gummy sugars in the fibers, which can trap water and be released to the DPC during the milling process [25]. These results are in agreement with the results of Arabshahi-Delouee et al. [56] for yogurt with flaxseed press cake, Karaca et al. [24] for yogurt with apricot press cake, Pérez-Chabela et al. [26] for yogurt with mango and potato peels powder, Rojas-Torres et al. [57] for yogurt with butternut squash, and Diep et al. [27] for yogurt with tamarillo.

Figure 3b display the effect of DPC treatments and storage period on the WHC. With the increase in DPC percent, the WHC values decreased during storage. For example, the WHC values for treatments T_0_, T_1_, T_2_, and T_3_ were 51.8, 56.76, 58.66, and 58.9 at the beginning of the storage period, while they were 47, 48.96, 52.1, and 54.13 at the end of the storage period (*p* < 0.05). These results are in agreement with the results of Güler-Akın et al. [58] for yogurt with oat and inulin fibers, Karaca et al. [24] for yogurt with apricot press cake, and Diep et al. [27] for yogurt with tamarillo.

**Figure 4:**
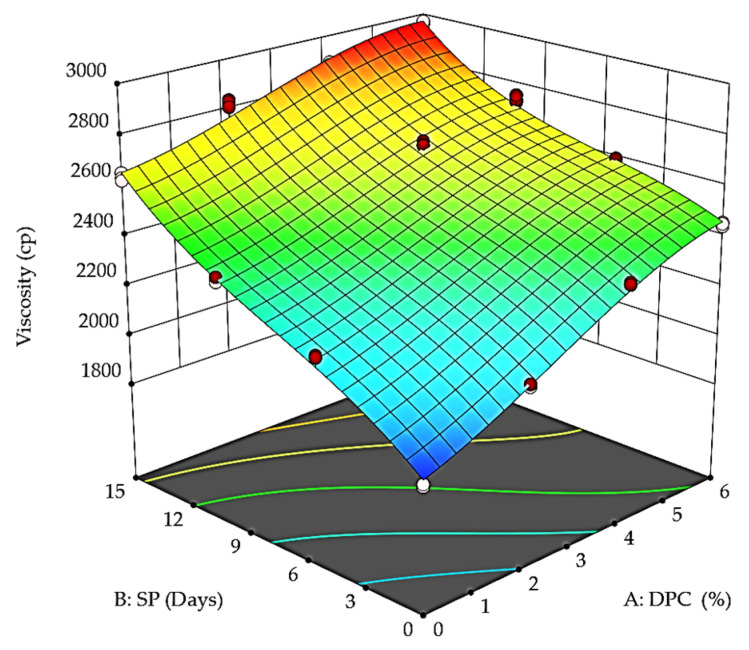
Effect of DPC percent (A: DPC) and storage period (B: SP) on the viscosity of DPC drinkable yogurt during the storage period at 4 °C.

The viscosity results (Figure 4) showed the highest values at the end of the storage period for all DPC yogurt treatments—T_1_, T_2_, and T_3_—compared to the control sample T_0_. In addition, the viscosity values increased directly with the DPC increase. For example, T_3_ recorded 2452.33 and 2962.66 cp at the beginning and end of the storage period, respectively. The findings are in agreement with the results of Karaca et al. [24] for yogurt with apricot press cake, Varnait et al. [6] for yogurt with blackberry press cake, and Diep et al. [27] for yogurt with tamarillo.

On the other hand, the results of the study conducted by Tseng et al. [59] found that the use of 3% of red grape peels after fermentation weakened the viscosity of yogurt. As the addition to yogurt takes place after or before the fermentation process, Cichonska et al. [33] stated that the statistical analysis of their results showed the differences in the viscosity of yogurt when milled flaxseed was added after fermentation and before fermentation compared to the control sample. The viscosity of yogurt significantly increased when ground flaxseeds were added after fermentation, while the viscosity decreased when they were added before fermentation.

#### 3.2.4. Color Parameters of DPC Drinkable Yogurt

**Figure 5:**
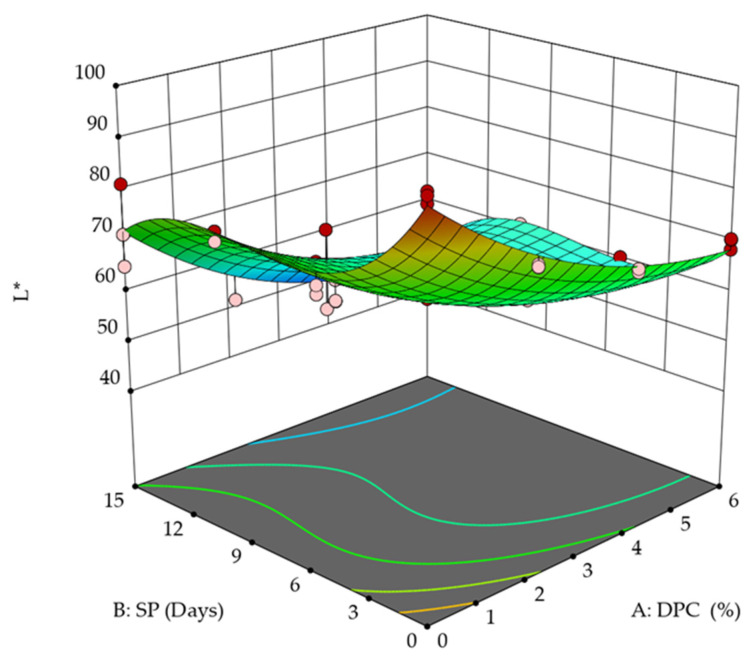
Effect of DPC addition percent (A: DPC) and storage period (B: SP) on L* of DPC drinkable yogurt treatments during the storage period at 4 °C.

**Figure 6:**
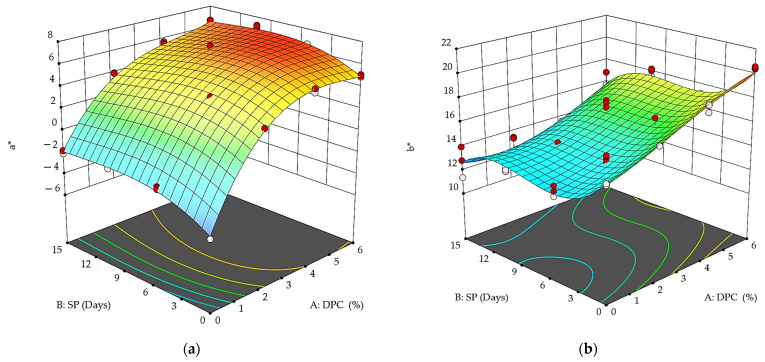
Effect of DPC addition percent (A: DPC) and storage period (B: SP) on a* and b* of DPC drinkable yogurt treatments during the storage period at 4 °C.

The color measurement value determinants given by Hunter Lab Device L*, a*, b* for DPC drinkable yogurt treatments during the storage period are shown in Figure 5 and Figure 6. The L* values ranged between 69.83 and 78.45 for DPC yogurt treatments vs. the control treatment of 96.75 in the first storage period (Figure 5). In addition, they ranged between 45.92 and 57.54 compared to the control treatment and 72.6 at the end of the storage period. T_3_ containing 6% of DPC was the darkest treatment according to the L* values, which amounted to 69.83 at the beginning of the storage period. The degrees of whiteness decreased significantly during the storage period for all treatments (*p* < 0.05). 

Regarding a*, the results indicated that the control sample during the first and last storage period was negative, ranging from −4.24 to −1.91, which indicated its tendency toward greenness, and the degree of greenness decreased at the end of the storage period compared to its beginning (Figure 6a). On the other hand, all DPC yogurt treatments—T_1,_ T_2,_ and T_3_—recorded significant (*p* < 0.05) positive values—3.3, 5.04, and 4.98, for T_1_, T_2,_ and T_3,_ respectively—at the beginning of the storage period, and the values were 3.69, 5.46, and 6.35 at the end of the storage period. This indicates a tendency toward redness, which may be attributed to the reddish-brown color of the DPC. Thus, the storage period significantly (*p* < 0.05) affected the a* values, as the values increased, as did their tendency to redden more at the end of the storage period compared to its beginning.

The b* values of DPC drinkable yogurt treatments revealed yellowing at the beginning and end of the storage period (Figure 6b). The control sample recorded significantly lower values (*p* < 0.05) than all treatments at the beginning and end of the storage period, whereby it scored 15.35 at the beginning of storage and 12.72 at the end of it. T_3_ was the most yellow, with 20.47, followed by T_2_ with 18.49 and T_1_ with 16.9, compared to the control T_0_, which reached 15.35 at the beginning of the storage period.

**Figure 7:**
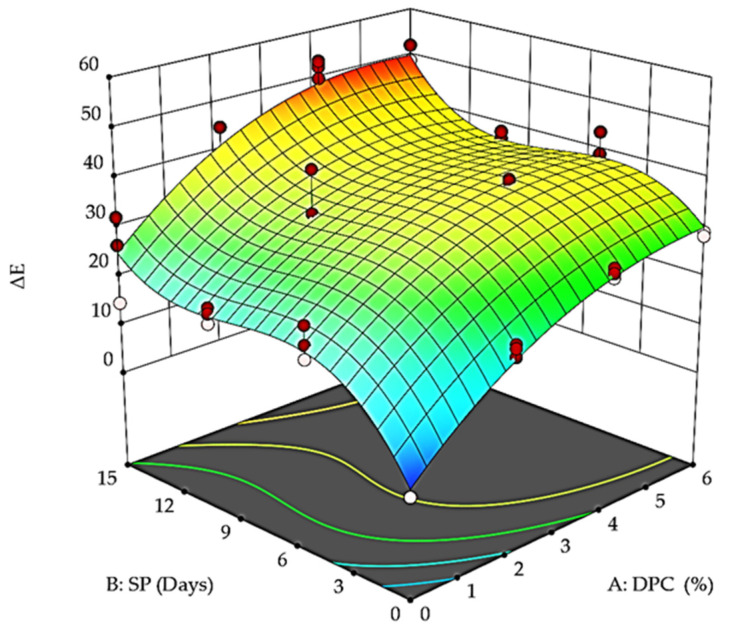
Effect of DPC addition percent (A: DPC) and storage period (B: SP) on the color difference (ΔE) value of DPC drinkable yogurt treatments during the storage period at 4 °C.

**Figure 8:**
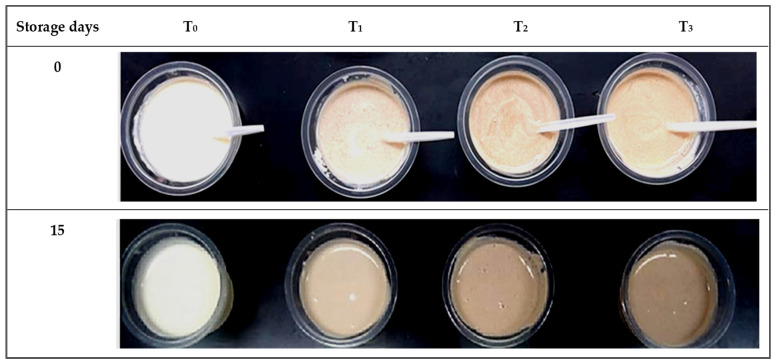
Visual color of DPC drinkable yogurt treatments during the storage period. T_0_, T_1_, T_2_, and T_3_ treatments refer to the percentage of DPC added to drinkable yogurt at 0, 2, 4, and 6%, respectively.

Regarding the color difference (ΔE) value, the results indicated that the samples in the first storage period were zero because this is the baseline (control), as shown in Figure 7, accompanied by the visual color at the beginning and end of the storage period (Figure 8). The highest values were found at the end of the storage period for all DPC drinkable yogurt treatments. In addition, ΔE values increased directly with increasing DPC percentage and storage period. For example, after five days, T_0_, T_1_, T_2_, and T_3,_ ΔE recorded 21.13, 30.7, 37.52, and 40.39. This did not change significantly subsequently for another 10 days, and then, values of 24.42, 40.1, 51.79, and 48.18, respectively, were recorded after 15 days. Generally, all DPC yogurt treatment values—T_1_, T_2_, and T_3_—were significantly (*p* < 0.05) decreased with increasing DPC and storage period at 4 °C. Figure 8 shows the visual color of DPC drinkable yogurt treatments during the storage period.

Rojas-Torres et al. [57] indicated that yogurt samples supplemented with thickeners from butternut squash seeds were affected in terms of glossiness, which they attributed to the size of the fat granules and protein. Łopusiewicz et al. [11] found that adding Camelina press cake to yogurt affected the a* (redness) value of the yogurt treatments, which the study attributed to the presence of a carotenoid pigment; however, the values decreased during the storage period due to the decrease in the stability of the dye.

Additionally, a decrease in a* (redness) values was observed in yogurt supplemented with flaxseed press cake as a result of fermentation [60]. In a study where ginseng root extract was added to yogurt, the results showed that the color values were affected by the addition, as the L* values decreased from 93.96 to 90.99, while a* and b* values increased from 2.92 and 2.30 to 5.91 and 11.11, respectively. Darkness and decreased luminosity L* compared to the control samples combined with increased b* values resulted in a yellowish color [55]. Alqahtani et al. [61] mentioned the effect of yogurt with tomato pomace on color measurements during storage. The results indicated a decrease in the whiteness values of L* and increases in the values of a* and b*. The results of Mkadem et al. [28] in enhancing the fermented milk drink with dried dates showed its effects on the color characteristics, especially a* and b*, where the a* value increased from −1.8 ± 0.8 to 4.5 ± 0.2, while the b* value increased from 3.1 ± 0.1 to 12.1 ± 0.1. In contrast, the value of the gloss L* decreased, which was attributed to the dyes of the date dryer. Adding dried tamarillo plant fiber (5–15%) to yogurt decreased the L* value, while the value of a* increased with the increase in the addition percentage [27]. The study attributed this to the natural pigments of the anthocyanins in tamarillo. In a study mixing microencapsulated guava pomace fibers with yogurt, the L* value was unaffected, and the values of a* and b* within the treatments were close; this was due to the contents of beta-carotene inside the guava pomace fibers used [62].

### 3.3. Correlation between the Factors and Physicochemical Properties of DPC Drinkable Yogurt

**Figure 9:**
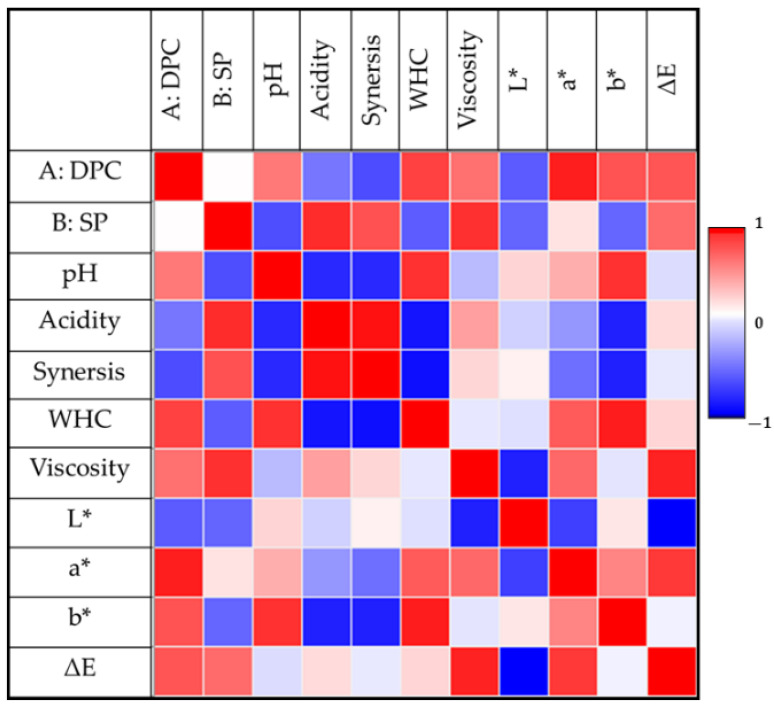
The correlation between the DPC treatments (A: DPC), storage period (B: SP), and physicochemical properties of DPC drinkable yogurt. WHC, L*, a*, b*, and ΔE, refers to water holding capacity, lightness, redness/greenness, yellowness/blueness, and color difference, respectively.

Figure 9 displays the correlation between the DPC treatments, storage period, and physicochemical properties of DPC drinkable yogurt. Significant positive correlations exist between the DPC treatments and pH, WHC, viscosity, a*, b*, and the DPC drinkable yogurt’s color difference (ΔE).

On the other hand, there is a significant negative correlation between the DPC treatments and acidity, syneresis, and L* value. Regarding the storage period, there is a significant positive correlation between the DPC treatments and acidity, syneresis, viscosity, and ΔE of the DPC drinkable yogurt and a significant negative correlation between the DPC treatments and pH, WHC, L*, and b* value of the DPC drinkable yogurt. In addition, a slight positive correlation existed between the a* value and storage period.

### 3.4. Modeling of Physicochemical Properties of DPC Drinkable Yogurt 

In order to model the physicochemical properties of DPC drinkable yogurt, the experiment outcomes were input into the Design Expert software for additional data analysis. The data of the two factors (DPC percent and storage period) were fitted with different models, i.e., linear, quadratic, and cubic, in order to model the physicochemical properties of DPC drinkable yogurt. The quadratic polynomial models most appropriately described the physicochemical properties of DPC drinkable yogurt, i.e., pH, acidity, syneresis, WHC, viscosity, and ΔE. The final predictive models for the two essential factors for pH, acidity, syneresis, WHC, viscosity, and ΔE are presented as Equations (5)–(13), respectively. These equations can be used to predict the mentioned physicochemical properties based on the input values for the actual percentage of the DPC and storage time (Day).

pH = 4.49 + 0.006 DPC − 0.011 SP − 0.001 DPC × SP + 0.001 DPC² + 0.001 SP²(5)

Acidity = 0.722 − 0.022 DPC + 0.007 SP + 0.001 DPC × SP + 0.001 DPC² + 0.001 SP²(6)

Syneresis = 50.892 − 1.698 DPC + 0.217 SP + 0.023 DPC × SP + 0.064 DPC² + 0.011 SP²(7)

WHC = 51.96517 + 2.237 DPC − 0.481 SP − 0.137400 DPC × SP + 0.001 DPC² + 0.025 SP²(8)

Viscosity = 1915.18 + 70.869 DPC + 68.915 SP − 5.233 DPC × SP + 16.398 DPC² − 4.497 SP²(9)

L* = 95.321 − 9.31 DPC − 7.189 SP + 0.374 DPC × SP + 0.97 DPC² + 0.891 SP²(10)

a* = −4.019 + 5.267 DPC + 0.61 SP − 0.081 DPC × SP − 0.977 DPC² − 0.051 SP²(11)

b* = 15.413 + 0.546 DPC − 1.108 SP − 0.068 DPC × SP + 0.144 DPC² 0.159 SP²(12)

ΔE = 1.514 + 10.344 DPC + 7.107 SP − 0.406 DPC × SP − 1.165 DPC² − 0.869 SP²(13)

where DPC is the date press cake percentage, and SP is the daily storage period.

The standard deviation (Std. Dev.), mean value, coefficient of variation percentage (C.V.%), coefficient of determination (R^2^), adjusted R^2^, and predicted R^2^ Adeq precision criteria were used to evaluate the selected predicted models (Equations (5)–(13)) in terms of the pH, acidity, syneresis, WHC, viscosity, L*, a*, b*, and ΔE of DPC drinkable yogurt. The quadratic model emerged as the best because it exhibited a low standard deviation, high R-squared values close to 1, and low PRESS. The evaluation criteria of the prediction models are shown in Table 3. The evaluation criteria indicated that the selected prediction models could efficiently describe the experiments. Therefore, these models can be used to navigate the design space for the target physicochemical properties of DPC drinkable yogurt responses. The results of the quadratic models agree with Mohammed et al. [63].

**Figure 10:**
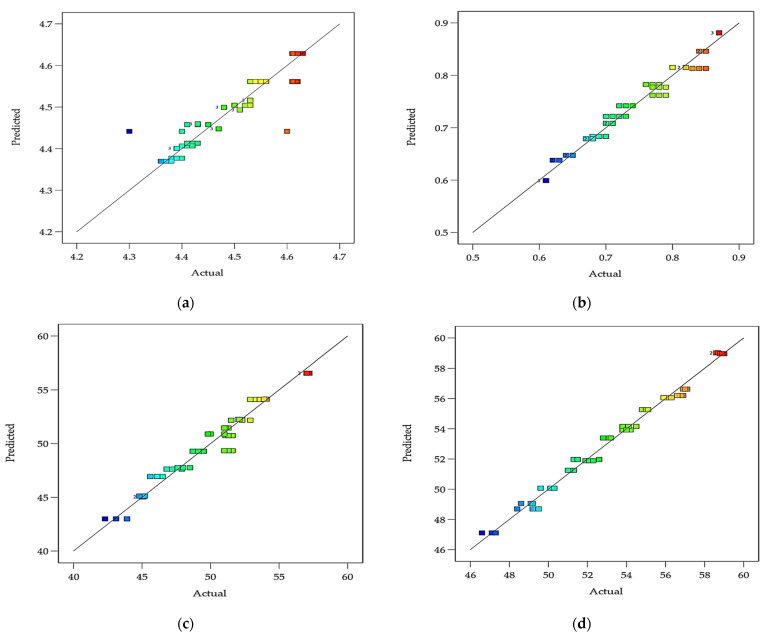

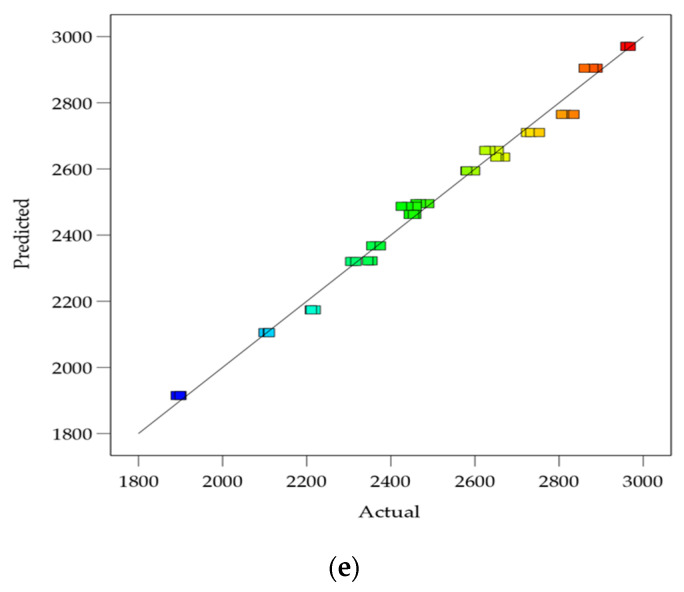
Scatter plots of the actual vs. predicted values generated by the prediction models of the pH (**a**), acidity (**b**), syneresis (**c**), WHC (**d**) and viscosity (**e**), and ΔE (**f**) of DPC drinkable yogurt.

**Figure 11:**
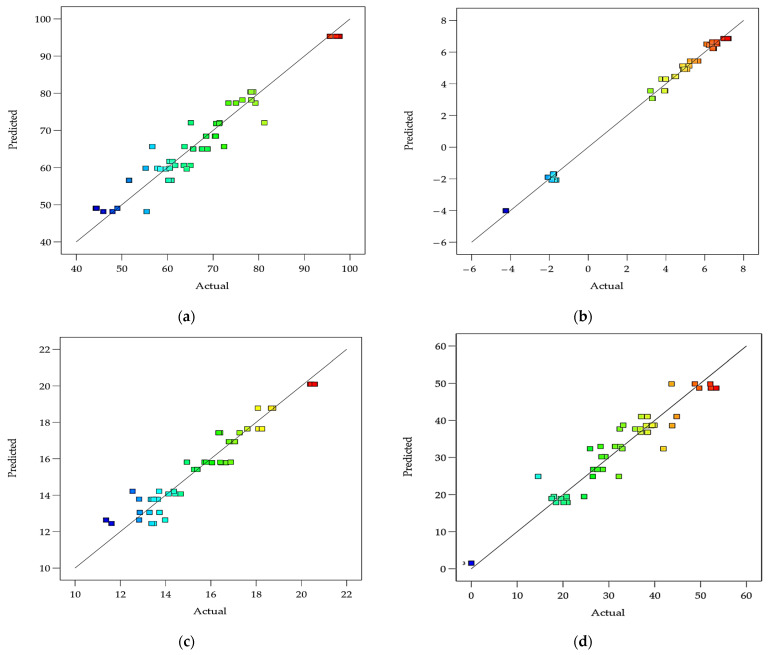
Scatter plots of the actual vs. predicted values generated by the prediction models of the L (**a**), a* (**b**), b* (**c**), ΔE (**d**) of DPC drinkable yogurt.

Figure 10a–e show the scatter plots of the predicted values generated by the developed models of the pH, acidity, syneresis, WHC, viscosity, and ΔE of DPC drinkable yogurt vs. the actual experimental values. Figure 11a–d show the scatter plots of the predicted values generated by the developed models of the L*, a*, b*, and ΔE of DPC drinkable yogurt vs. the actual experimental values. The results indicated that the predicted values were close to the observed values. Therefore, the prediction model is suitable for responses to the target physicochemical properties of DPC drinkable yogurt.

### 3.5. Texture Profile Analysis of DPC Drinkable Yogurt

The texture profile analysis results (Table 4) indicated that hardness (g) increased with the increase in the addition of DPC, and sample T_3_ recorded a value of 491.83 compared to 316.23 for the control sample at the beginning of the storage period, while the value was 581.33 compared to 506.6 for the control sample at the end of the storage period. On the other hand, the adhesiveness of treatments decreased with an increase in the addition of DPC (negative increasing numbers), with treatments T_3_ and T_2_ obtaining the lowest scores of −368 and −371.33 compared with sample T_1_, which scored −349, and the control sample, which scored −286 at the beginning of storage. The values at the end of storage for the same samples were −455.33, −453.33, −445, and 411.33, respectively.

Although the springiness values increased with the increase in DPC, the increase was significantly inversely proportional (*p* < 0.05) to the progression of the storage period. Treatment T_3_ recorded the highest value of 0.93 vs. 0.83 for the control sample at the beginning of the storage period, and the value was 0.79 vs. 0.71 for the control sample at the end of the storage period. This approach to characterization of springiness values was followed for each of the values of cohesiveness and gumminess. At the same time, the resilience values decreased with the increase in the addition of DPC and the progression of the storage period, with treatment T_3_ recording a value of 0.31 vs. 0.64 for the control sample at the beginning of the storage period and 0.1 vs. 0.51 for the control sample at the end of the storage period. The results of the texture profile analysis in this study agree with those in previous studies [24,36,44,61,62,64].

### 3.6. Sensory Evaluation of DPC Drinkable Yogurt 

**Figure 12:**
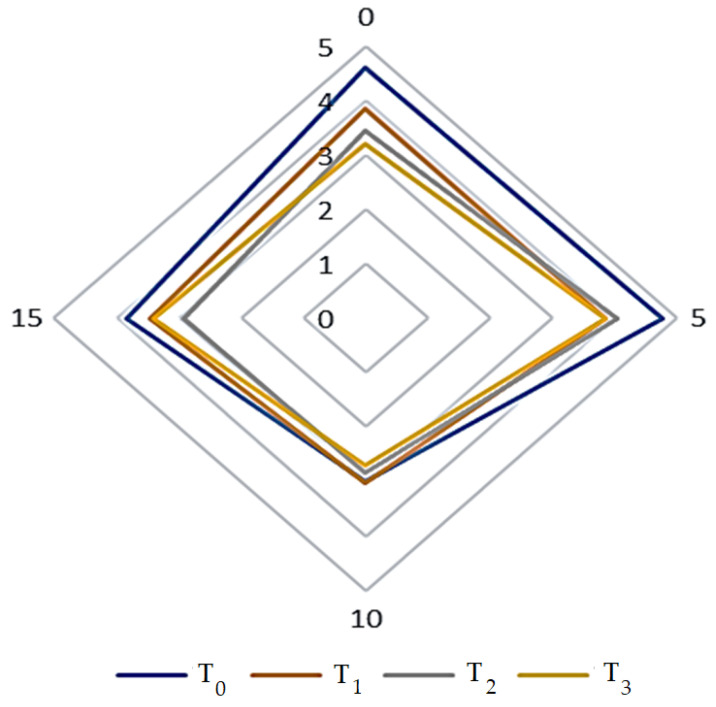
Overall acceptability of DPC drinkable yogurt treatments during storage period at 4 °C. Treatments T_0_, T_1_, T_2_, and T_3_ refer to the percentage of DPC added to drinkable yogurt at 0, 2, 4, and 6%, respectively.

The results of sensory acceptance regarding the color, texture, flavor, and general acceptance of DPC drinkable yogurt during the storage period at 4 °C showed that samples T_1_ followed by T_2_ and T_3_ were the closest in overall sensory traits to the control sample (*p* < 0.05) over the progression of the storage period (Table 5 and Figure 12). T_1_ recorded values of sensory preference at the beginning of the storage period in terms of color, texture, flavor, and general acceptance of 4, 3.76, 3.73, and 3.86 vs. the control sample with 4.3, 4, 4.1, and 4.26; at the end of the storage period, it reached values of 3.66, 3.73, 3.53, and 3.46 vs. the control sample with 4.30, 3.90, 3.80, and 3.83, respectively. These results are consistent with Łopusiewicz et al. [11]—who used Camelina press cake with yogurt—regarding the color effect. Karaca et al. [24] stated that the increase in the addition of apricot press cake fibers affected the arbitrators’ preference for yogurt samples due to the increase in viscosity. At the same time, the results differed in terms of the effect of the storage period. Brodziak et al. [65] mentioned that adding sea buckthorn fruit to yogurt affected the organoleptic characteristics of addition and storage. The sensory characteristics were improved in yogurt samples with thickeners added from butternut squash seeds [57]. In another study [55] attempting to enhance yogurt with hydroponic ginseng extracts, the results showed that the treatment in terms of color was superior to the control sample at 1% concentration. At the same time, the preference decreased with increasing concentrations of it. At the same time, the texture and flavor were not affected by the addition of ginseng. With the addition of moringa to yogurt, the results of the study mentioned by Mendoza-Taco et al. [66] showed that the yogurt samples were not affected in relation to the sensory traits assessed. Meanwhile, in a study [67] attempting to enhance the vitality-boosting fermented camel milk drink with sugary date fibers and demonstrate its effects on sensory traits, the results indicated that adding 12.5% of sugary dates fiber was preferred by sensory traits assessors. 

The addition of 0.5% of dried orange peel fibers did not have any significant effects (*p* < 0.05) on the sensory acceptance of the viability-boosting yogurt drink treatments compared to the control sample, while increasing the concentrations of dried orange peel fibers up to 2% had a negative effect on the sensory properties [68].

## 4. Conclusions

Adding DPC powder before the fermentation of DPC yogurt slightly increased the pH and decreased acidity during the storage period. DPC addition slightly reduced syneresis values and increased the WHC during storage. Viscosity recorded the highest value at the end of the storage period for all treatments. Yogurt containing 6% DPC was the highest in viscosity. The L* values decreased significantly (*p* < 0.05) with increasing DPC, and the degrees of whiteness or glossiness decreased significantly during the storage period for all treatments. The a* values were positive for all DPC yogurt samples, and the b* values showed yellowness in all DPC treatments at the beginning and end of the storage. Texture profile data indicated that increased DPC and the storage period’s progression increased the hardness (g), springiness, cohesiveness, and gumminess. Adding DPC reduced adhesiveness and resilience during storage. Yogurt containing 2% was the closest in overall sensory acceptability to the control sample (*p* < 0.05) and during the storage. The predicted values of the quadratic models were close to the actual observed values for the pH, acidity, syneresis, WHC, viscosity, and ΔE of the treated DPC drinkable yogurt. Generally, the results indicated the possibility of enriching yogurt drinks by 2–4% with DPC as an innovative functional additive with general acceptance. The results also support the idea of expanding the uses of DPC in yogurt, enhancing the circular economy for waste upgrading, and DPC can be proposed as a new functional fiber-based yogurt. Overall, these results gave a new idea regarding several aspects related to the upcycling vision, ranging from technological strategies to reusing agrifood by-products and obtaining functional ingredients for high-value-added food production. The sensory evaluation may require more participants in a further study before the proposed product can be applied on a large scale.

## Figures and Tables

**Table 1:** Physicochemical analysis of milk used and DPC.

Materials	Properties	Values
Milk	TS (g/100 g)	11.19 ± 0.11
	Protein (g/100 g)	2.97 ± 0.02
	Fat (g/100 g)	2.26 ± 0.05
	Ash (g/100 g)	0.68 ± 0.01
	pH	6.76 ± 0.04
DPC	Moisture (g/100 g)	7.40 ± 0.12
	Protein (g/100 g)	4.52 ± 0.21
	Fat (g/100 g)	2.69 ± 0.03
	Ash (g/100 g)	2.26 ± 0.15
	Crude fiber (g/100 g)	8.36 ± 0.11
	* Total saccharides (g/100 g)	79.13 ± 2.35
	WHC (g/100 g)	3.49 ± 0.11
	Solubility (g/100 g)	15.46 ± 0.42
	pH	5.32 ± 0.05
	L*	64.52 ± 8.79
	a*	11.19 ± 1.20
	b*	27.28 ± 3.14

The indicative values of the parameters are the means (±SD) of three replicates. * Calculated according to the equation = 100 – (moisture + protein + fat + ash).

**Table 2:** Physicochemical analysis of DPC drinkable yogurt treatments during storage period at 4 °C.

Treatments	Total Solids (g/100 g)	Protein (g/100 g)	Fat (g/100 g)
T_0_	11.19 ± 0.11 ^d^	2.97 ± 0.02 ^d^	2.26 ± 0.05 ^b^
T_1_	11.83 ± 0.03 ^c^	3.10 ± 0.10 ^c^	2.34 ± 0.01 ^a^
T_2_	12.92 ± 0.05 ^b^	3.30 ± 0.04 ^b^	2.34 ± 0.02 ^a^
T_3_	13.45 ± 0.13 ^a^	3.42 ± 0.01 ^a^	2.35 ± 0.02 ^a^

The indicative values of the parameters are the means (±SD) of three replicates. Lowercase letters a, b, c, and d for the horizontal comparison between the treatments and the significance of the difference within (*p* < 0.05) limits. T_0_, T_1_, T_2_, and T_3_ treatments refer to the percentage of DPC added to drinkable yogurt at 0, 2, 4, and 6%, respectively.

**Table 3:** The evaluation criteria, i.e., standard deviation (Std. Dev.), mean value, coefficient of variation percentage (C.V.%), coefficient of determination (R^2^), adjusted R^2^, predicted R^2^, and Adeq precision criteria, for the selected quadratic models for the target physicochemical properties of DPC drinkable yogurt.

Criteria	Physicochemical Properties of DPC Drinkable Yogurt								
	pH	Acidity	Syneresis	WHC	Viscosity	L*	a*	b*	ΔE
Std. Dev.	0.040	0.014	0.783	0.405	30.85	3.916	0.264	0.706	3.99
Mean	4.47	0.738	49.77	53.35	2492.85	66.86	3.364	15.48	31.02
C.V.%	0.894	1.86	1.57	0.759	1.24	5.858	7.875	4.565	12.85
R²	0.779	0.973	0.961	0.989	0.990	0.919	0.995	0.922	0.921
Adjusted R²	0.752	0.969	0.956	0.987	0.988	0.868	0.994	0.905	0.902
Predicted R²	0.717	0.964	0.949	0.983	0.985	23.78	0.993	0.874	0.870
Adeq Precision	18.316	58.098	48.999	64.421	74.943	3.916	89.907	23.717	26.563

WHC, L*, a*, b*, and ΔE, refers to water holding capacity, lightness, redness/greenness, yellow-ness/blueness, and color difference, respectively.

**Table 4:** Texture profile analysis of DPC drinkable yogurt treatments during storage.

Texture Parameters	Storage Days	T_0_	T_1_	T_2_	T_3_
Hardness, g	0	316.23 ± 8.28 ^Ad^	432.73 ± 2.51 ^Bc^	451.83 ± 1.60 ^Bb^	491.83 ± 3.68 ^Ba^
	15	506.60 ± 7.93 ^Bd^	524.50 ± 9.35 ^Ac^	547.76 ± 4.28 ^Ab^	581.33 ± 2.06 ^Aa^
Adhesiveness, g s	0	−286.00 ± 4.35 ^Aa^	−349.00 ± 8.54 ^Ab^	−371.33 ± 1.52 ^Ac^	−368.00 ± 5.29 ^Ac^
	15	−411.33 ± 1.52 ^Ba^	−445.00 ± 6.08 ^Bb^	−453.33 ± 2.51 ^Bc^	−455.33 ± 5.03 ^Bc^
Springiness	0	0.83 ± 0.00 ^Ac^	0.86 ± 0.02 ^Ab^	0.92 ± 0.01 ^Aa^	0.93 ± 0.005 ^Aa^
	15	0.71 ± 0.005 ^Bb^	0.73 ± 0.01 ^Bb^	0.75 ± 0.04 ^Bab^	0.79 ± 0.01 ^Ba^
Cohesiveness	0	0.80 ± 0.005 ^Ad^	0.84 ± 0.005 ^Ac^	0.86 ± 0.005 ^Ab^	0.97 ± 0.01 ^Aa^
	15	0.54 ± 0.04 ^Bb^	0.57 ± 0.01 ^Bb^	0.70 ± 0.005 ^Ba^	0.73 ± 0.02 ^Ba^
Gumminess	0	399.10 ± 0.85 ^Ab^	142.23 ± 0.32 ^Bd^	367.66 ± 0.49 ^Ac^	578.56 ± 0.51 ^Aa^
	15	282.43–0.30 ^Bc^	244.80 ± 0.26 ^Ad^	366.5 ± 0.50 ^Ba^	291.16 ± 1.04 ^Bb^
Resilience	0	0.64 ± 0.02 ^Aa^	0.52 ± 0.02 ^Ab^	0.55 ± 0.04 ^Ab^	0.31 ± 0.01 ^Ac^
	15	0.53 ± 0.02 ^Ba^	0.34 ± 0.04 ^Bb^	0.15 ± 0.04 ^Bc^	0.10 ± 0.01 ^Bc^

The indicative values of the parameters are the means (±SD) of three replicates. The uppercase letters A, B, C, and D represent the vertical comparison between storage periods. The lowercase letters a, b, c, and d represent the horizontal comparison between the treatments. Treatment T_0_, T_1_, T_2_, and T_3_ refers to the percentage of DPC added to drinkable yogurt at 0, 2, 4, and 6%, respectively.

**Table 5:** Sensory evaluation of DPC drinkable yogurt treatments during storage period at 4 °C.

Sensory Parameters	Storage Days	T_0_	T_1_	T_2_	T_3_
Color	0	4.30 ± 1.34 ^Aa^	4.00 ± 1.08 ^ABa^	3.20 ± 0.88 ^Bb^	2.90 ± 1.26 ^Bb^
	5	4.70 ± 0.74 ^Aa^	4.26 ± 0.94 ^Aab^	4.40 ± 0.85 ^Aab^	4.16 ± 1.01 ^Ab^
	10	3.80 ± 0.84 ^Ba^	3.53 ± 0.77 ^Cab^	3.43 ± 0.72 ^Bab^	3.16 ± 0.98 ^Bb^
	15	4.30–0.59 ^Aa^	3.66 ± 0.66 ^BCbc^	3.26 ± 1.04 ^Bc^	3.80 ± 0.80 ^Ab^
Texture	0	4.00 ± 1.28 ^Ba^	3.76 ± 1.16 ^Aab^	3.46 ± 1.10 ^ABab^	3.26 ± 1.11 ^Bb^
	5	4.63 ± 0.55 ^Aa^	3.86 ± 1.04 ^Ab^	3.70 ± 1.11 ^Ab^	3.70 ± 1.41 ^ABb^
	10	3.50 ± 0.86 ^Ca^	3.50 ± 0.97 ^Aa^	3.03 ± 0.88 ^Ba^	3.23 ± 1.00 ^Ba^
	15	3.90 ± 0.66 ^BCa^	3.73 ± 0.90 ^Aa^	3.10 ± 0.92 ^Bb^	3.86 ± 1.10 ^Aa^
Flavor	0	4.10 ± 1.29 ^Ba^	3.73 ± 1.31 ^Aab^	3.10 ± 1.29 ^Bbc^	3.00 ± 1.48 ^Bc^
	5	4.56 ± 0.62 ^Aa^	3.80 ± 1.39 ^Ab^	4.10 ± 0.88 ^Aab^	3.90 ± 1.34 ^Ab^
	10	2.96 ± 0.92 ^Ca^	3.10 ± 0.88 ^Ba^	2.90 ± 0.99 ^Ba^	2.76 ± 1.04 ^Ba^
	15	3.80 ± 0.61 ^Ba^	3.53 ± 0.93 ^ABab^	3.03 ± 1.12 ^Bb^	3.40 ± 1.19 ^ABab^

The indicative values of the parameters are the means (±SD) of three replicates. The uppercase letters A, B, C, and D represent the vertical comparison between storage periods, while the lowercase letters a, b, c, and d represent the horizontal comparison between the treatments, and the difference is significant within (*p* < 0.05). Treatment T_0_, T_1_, T_2_, and T_3_ refers to the percentage of DPC added to drinkable yogurt at 0, 2, 4, and 6%, respectively.

## Data Availability

Data are contained within the article.
